# Analysis of transient membrane protein interactions by single-molecule diffusional mobility shift assay

**DOI:** 10.1038/s12276-021-00567-1

**Published:** 2021-02-19

**Authors:** Min Gyu Jeong, Kai Zhou, Soyeon Park, HyeongJeon An, Yonghoon Kwon, Yeonho Chang, Do-Hyeon Kim, Sung Ho Ryu

**Affiliations:** 1 Department of Life Sciences, Pohang University of Science and Technology, Pohang, Republic of Korea; 2 Department of Physics, Pohang University of Science and Technology, Pohang, Republic of Korea

**Keywords:** Fluorescence imaging, Super-resolution microscopy, Single-molecule biophysics

## Abstract

Various repertoires of membrane protein interactions determine cellular responses to diverse environments around cells dynamically in space and time. Current assays, however, have limitations in unraveling these interactions in the physiological states in a living cell due to the lack of capability to probe the transient nature of these interactions on the crowded membrane. Here, we present a simple and robust assay that enables the investigation of transient protein interactions in living cells by using the single-molecule diffusional mobility shift assay (smDIMSA). Utilizing smDIMSA, we uncovered the interaction profile of EGFR with various membrane proteins and demonstrated the promiscuity of these interactions depending on the cancer cell line. The transient interaction profile obtained by smDIMSA will provide critical information to comprehend the crosstalk among various receptors on the plasma membrane.

## Cell membrane: assaying protein interactions in cancer cells

A new method for analyzing transient interactions between proteins in the outer membrane of living cells could help researchers better understand how cancer cells develop resistance to targeted drug therapies. A research team led by Do-Hyeon Kim and Sung Ho Ryu from Pohang University of Science and Technology, South Korea have developed a technique for investigating weak protein–protein interactions on the basis of changes in their rate of diffusion when bound to each other. Using this “single-molecule diffusional mobility shift assay,” the researchers showed that a cancer-linked protein called EGFR tends to bind with a range of other proteins, depending on the type of cancer cell studied. EGFR is the target of several drug therapies, and the variability of its interactions may account for its ability to circumvent drug-induced inhibition in resistant cancer cells.

## Introduction

Membrane proteins fulfill crucial roles in communication between the extracellular and intracellular environments across the plasma membrane through their dynamic interactions^1^. More than half of cellular proteins are involved in these interactions simultaneously in space and time, which determines cellular fates based on the diversity of the environments surrounding a cell. Yet not much of these interactions have been revealed^2,3^. Furthermore, the cell-to-cell or molecule-to-molecule heterogeneity of these interactions is barely known^4,5^. To elucidate these interactions, various assays and methodologies have been developed.

Biochemical assays for membrane protein interactions, such as co-immunoprecipitation and enzyme-linked immunosorbent assays, require purification of membrane proteins, which often results in the loss of genuine membrane protein interactions^6,7^. Genetic assays, such as the protein-fragment complementation assay and membrane-based yeast two-hybrid systems, show a high false-positive rate and typically measure only static interactions^8,9^. Fluorescence resonance energy transfer can measure dynamic interactions but has a structural limitation in that the two probes have to be located within several nanometers of each other. Fluorescence imaging assays, such as fluorescence recovery after photobleaching and fluorescence correlation spectroscopy, can mainly detect clustering or immobilization of membrane proteins^10,11^.

Recently, stochastic super-resolution techniques, such as photoactivated localization microscopy (PALM), stochastic optical reconstruction microscopy^12^, and DNA point accumulation in nanoscale topology, have achieved molecular localization on a scale of up to several nanometers^13–15^. These techniques have been applied for visualizing molecular localization on the plasma membrane, but they provide only limited information about protein interactions^16–18^.

However, none of these methods is viable for investigating physiological membrane protein interactions, because they cannot detect the transient nature of these interactions on the crowded membrane. Here, we present a platform assay for investigating transient protein interactions in living cells via single-molecule diffusional mobility shift assay (smDIMSA)^19^, which sensitively detects changes in individual proteins’ diffusivity induced by the binding of soluble proteins. Using smDIMSA, we revealed the transient interaction profile of EGFR in various cancer cell lines, which demonstrated an unprecedented level of promiscuity in its interactions in a manner dependent on the cell line.

## Materials and methods

### Cell culture and transfection

COS7, A431, and HeLa cells obtained from the American Type Culture Collection (ATCC) were cultured in Dulbecco’s modified Eagle’s medium (DMEM, Lonza) supplemented with 10% FBS (Gibco) at 37 °C in an environment of 5% CO_2_, and 95% humidity. A549 cells (ATCC) were cultured in DMEM/F-12 1:1 modified medium (Thermo) supplemented with 10% FBS (Gibco) at 37 °C in 5% CO_2_. Caco-2 cells (ATCC) were cultured in Eagle’s minimum essential medium (Thermo) supplemented with 20% FBS (Gibco) at 37 °C in 5% CO_2_. Skbr3 and Skov3 cells (ATCC) were cultured in McCoy’s 5A (modified) medium (Gibco) supplemented with 10% FBS (Gibco) at 37 °C in 5% CO_2_. Cells were transfected with Lipofectamine 2000 (Invitrogen) according to the manufacturer’s instructions.

### Plasmids, antibodies, and reagents

pcDNA3/YFP-CD28 and pcDNA3/YFP-CD86 were kind gifts from Dr. Moritz Bünemann at the University of Würzburg. As reported in their work, CD28 was truncated at R185, thereby deleting the cytosolic region, and CD86 was truncated at R227^20^. To track single molecules of CD28 and CD86, YFP was replaced with monomeric mEos3.2. To preserve the original signal peptide from pcDNA3/YFP-CD28, extension PCR was used: the mEos3.2 gene was amplified and extended with two primers (5′-CTACATCTTCTGCCTGGTATTCGCCATGAGTGCGATTAAGCCAGAC (forward) and 5′-GCTCTAGATCGTCTGGCATTGTCAGGCAA (reverse)) and a subsequent PCR extension step with the primers 5′-GGATCCATGAAGACGATCATCGCCCTGAGCTACATCTTCTGCCTGG (forward, underline: BamHI site) and 5′-GCTCTAGATCGTCTGGCATTGTCAGGCAA (reverse, underline: XbaI site). The resulting product was cloned into pcDNA3/YFP-CD28 or pcDNA3/YFP-CD86 to generate pcDNA3/mEos3.2-CD28 and pcDNA3/mEos3.2-CD86 using BamHI and XbaI, respectively. EGFR WT-mEos3.2 was constructed as previously reported^19^.

Monoclonal antibodies against the extracellular regions of CD86 and CD28 were obtained from R&D Systems (MAB141-100 and MAB342-100, respectively). The monoclonal antibody against the N-terminus of EGFR was purchased from Thermo Scientific (clone 199.12, #MS-396-P1ABX). Monoclonal antibodies against ErbB2 and ErbB3 were purchased from EMD Millipore (OP39 and OP119, respectively). The monoclonal antibody against MET was obtained from Cell Signaling (8741). Antibodies against CD44, E-cadherin, and TLR1 were obtained from Abcam (ab9524, ab40772, and ab11209, respectively). The monoclonal antibody against integrin5a was obtained from R&D Systems (MAB1864). Two antibodies against EPHA2 were obtained—one from Sigma (WH0005159M8) and one from Abcam (ab73254). Two antibodies against PDGFRb were obtained—one from Sigma (WH0001969M1) and one from R&D Systems (MAB385). IgG was obtained from Abcam (ab6708). Fab fragments of anti-CD28, anti-CD86, and anti-EGFR antibodies were prepared with a Fab preparation kit (44685, Pierce) and purified using size exclusion chromatography. The purified Fab fragments were then conjugated with Alexa Fluor 647 using an Alexa Fluor^®^ 647 Antibody Labeling Kit (Life Technologies, A-20186). For photoactivation of Alexa Fluor 647, protocatechuate-3,4-dioxygenase (PCD; P8279, Sigma-Aldrich) and β-mercaptoethylamine (MEA; 30070, Sigma-Aldrich) were used.

The selection of neutral antibodies was performed using established criteria. The criteria that we established were as follows: the antibody needed to be monoclonal and target the extracellular domain, yet the target in the extracellular domain needed to be far away from the dimerization arm or the domain that included the dimerization arm and needed to be functionally neutral to avoid disruption of the interaction status. While information about the monoclonality and extracellular domain-targeting properties of antibodies is provided by most antibody vendors, information about the exact domain that the antibody targets or the functional neutrality of antibodies is rarely provided. Therefore, for antibodies for which such information was not provided, we ordered many different antibodies from different companies to assess whether each individual antibody disrupts the interaction status. The selected antibodies were then evaluated to determine whether they caused a shift in the diffusion coefficient of the target receptor by genetically tagging the target receptor with the fluorescent protein mEos3.2 and visualizing the receptor before and after treatment with the antibody (Supplementary Fig. 1).

### Imaging sample preparation

Coverslips were cleaned with acetone for 30 min at 42 °C, rinsed with Milli-Q water three times, and etched with 1% hydrofluoric acid for 10 min at 42 °C. The coverslips were then extensively washed with Milli-Q water and stored in ethanol.

Before cell seeding, the coverslips were coated with fibronectin (10 μg/mL) for 30 min. Transfected cells with different expression levels of various receptors were enriched using a cell sorter (MoFlo^TM^ XDP, Beckman Coulter) when necessary. Phenol red was excluded from the medium for seeded cells to reduce cellular autofluorescence.

### Optical setup and image acquisition

Single-molecule imaging was performed with a custom-made objective based on total internal reflection fluorescence microscopy. The inverted microscope system used was from Olympus (IX-71) and was connected to a live-cell imaging chamber (Chamlide TC-A, Live Cell Instrument) capable of maintaining an environment of 37 °C and 5% CO_2_. The imaging chamber was mounted on an automated stage control system (MS-2000, Applied Scientific Instrumentation). Four laser beams of different wavelengths (405 nm, DL-405-100 from Crystal Laser; 488 nm, 400-A02-543 from Melles Griot; 561 nm, YLK-561-50-0.1-1 from Lasos; and 642 nm, 2RU-VFL-P-1500-647-B1R from MBP Communications) were collimated and focused into the rear port of the microscope after a beam expander (GBR03-A, ThorLabs) and a translation stage to control TIRF or epi-illumination. The lasers were focused with a 100X oil immersion objective (APON 100XOTIRF/1.49, Olympus). Laser illumination was controlled by mechanical shutters and a three-channel shutter driver (CS25S1T0 and VMM-D3, Uniblitz Electronic). For single-molecule imaging of mEos3.2, a dichroic mirror (ztuv-405-488-561rpc, Chroma) and an emission filter (zet405-488-561m, Semrock) were used. Typically, the intensity of the 561-nm laser was kept at 15 W cm^−2^, and the power of the 405-nm activation laser was dynamically varied according to the mEos3.2 expression level. For single-molecule imaging of Alexa Fluor 647, a dichroic mirror (zt405-488-561-647rpc, Chroma) and an emission filter (zet405-488-561-640m, Chroma) were used. The power of the 647-nm laser was set to 10 W cm^−2^. For two-color imaging, a beam splitter was placed within the TuCam system. For 488/561 imaging, a dichroic mirror (ET525/50 mm, Chroma) and two emission filters (T550lpxr and ET575lp, Chroma) were used. For 488/647 imaging, a dichroic mirror (ET535/70 m, Chroma) and two emission filters (T635lpxr and ET655lp, Chroma) were used. The sptPALM imaging scheme was similar to that previously reported^21^. Images were recorded at a frame rate of 19.2 Hz with two electron multiplying charge-coupled device cameras (iXon3 897, Andor Technology) connected to Andor’s TuCam system installed at the side port of the microscope. Data were acquired using MetaMorph software (molecular devices).

### Data analysis

Single-molecule detection and multiple particle tracking were performed as previously reported^19^. For each trajectory, the mean square displacement (MSD) was calculated as MSD(Δ*t*) = *E*[(*x*_*t* + Δ*t*_ − *x*_*t*_)^2^ + (*y*_*t* + Δ*t*_ − *y*_*t*_)^2^] with a time lag of four, where (*x*_*t*_, *y*_*t*_) are the Cartesian coordinates of the particle at time *t*. The MSD of most trajectories (>80%) followed a free diffusion mode^21^. Hence, the diffusion coefficient (D) of trajectories was determined using the following equation: MSD(Δ*t*) = 4D Δ*t* + 4*e*^2^, where *e* represents uncertainties accounting for localization error. Trajectories over eight frames were subjected to investigation.

To determine the changes in the diffusion coefficient before and after antibody treatment, a histogram showing the diffusion coefficient distribution for all molecules imaged was generated with a bin number set as the square root of the total number of molecules. The trajectory density did not change significantly before and after antibody treatment. Since we aimed to probe the dimerization of receptor tyrosine kinases (RTKs), we selected the mobile diffusional state, which is composed of monomers or small complexes capable of dimerization. We utilized the peak value of the logarithmic distribution of *D* for the fast population, which followed a log-normal distribution. The peak value of the diffusion coefficient of the fast and mobile population was determined with a weighted log-Gaussian fitting. The diffusion coefficient shift (*S*) was then calculated as *S* = (1 − *D*_p2_/*D*_p1_) × 100%. Here, *D*_p1_ and *D*_p2_ are the peak of diffusion coefficients before and after antibody treatment, respectively. All analyses were performed with custom-made codes in MATLAB. Multiple particle tracking was performed based on the U-track package^22^.

### Dimerization measurement

To measure receptor dimerization, membrane proteins were first partially labeled with an Alexa Fluor 647-conjugated Fab fragment of the indicated monoclonal antibody for 15 min. The Fab concentration for CD28 and CD86 was 20 nM. For the Fab fragment of mAb 199.12 specific for EGFR, the concentration was 8 nM. The labeled cells were washed with regular medium three times. Then, the cells were maintained in medium containing PCA (2.5 mM), PCD (0.2 U/mL), and MEA (1 mM) and imaged. After imaging under basal conditions, medium containing PCA, PCD, MEA, and the corresponding monoclonal antibodies were used to treat the cells for 15 min. The cells were then imaged again. Homodimer formation was evaluated by analyzing the diffusion coefficients of receptors before and after antibody treatment. To assess the effect of PIP_2_ on predimer formation of EGFR, cells were pretreated with DMSO (control, 0.1%), wortmannin (2 μM), or U73122/U73343 (1 μM) for 30 min. The same concentration of reagent was used for labeling and imaging. For measurement of EGF-induced EGFR dimerization, before labeling and imaging, cells were pretreated with erlotinib (5 μM) for 5 min and were then cotreated with erlotinib (5 μM) and EGF (1 nM) for 15 min. The other procedures were the same as those used for predimer measurement.

To determine the membrane density of EGFR, mEos3.2 was first bleached with the 488-nm laser to generate single molecules. Single-molecule images of the green form of mEos3.2 (488-nm channel) and the red form of mEos3.2 (561-nm channel) were recorded to calibrate the intensity. After single-particle tracking (Alexa Fluor 647-Fab) of individual cells, the green form of mEos3.2 (TIRF image) and the red form of mEos3.2 (single-molecule imaging with low 405 nm laser activation) were imaged with the same laser power and TIRF angle used for calibration. Then, the EGFR surface density was calculated as ${\mathrm{Density}}\,\left( {0.01\,{{\upmu}{\mathrm{m}}}^{ - 2}} \right) = \frac{{I_{488}/I_{561}}}{{I_{488s}^\prime /I_{561}^\prime }}$. Here, *I*_488_ is the average pixel intensity of the green form of mEos3.2, and *I*_561_ is the single-molecule intensity of red form of mEos3.2. *I*_488′_ and *I*_561′_ are the single-molecule intensities of the green and red forms, respectively, from the calibration.

## Results

### Concept of smDIMSA for the identification of protein–protein interactions

**Fig. 1:**
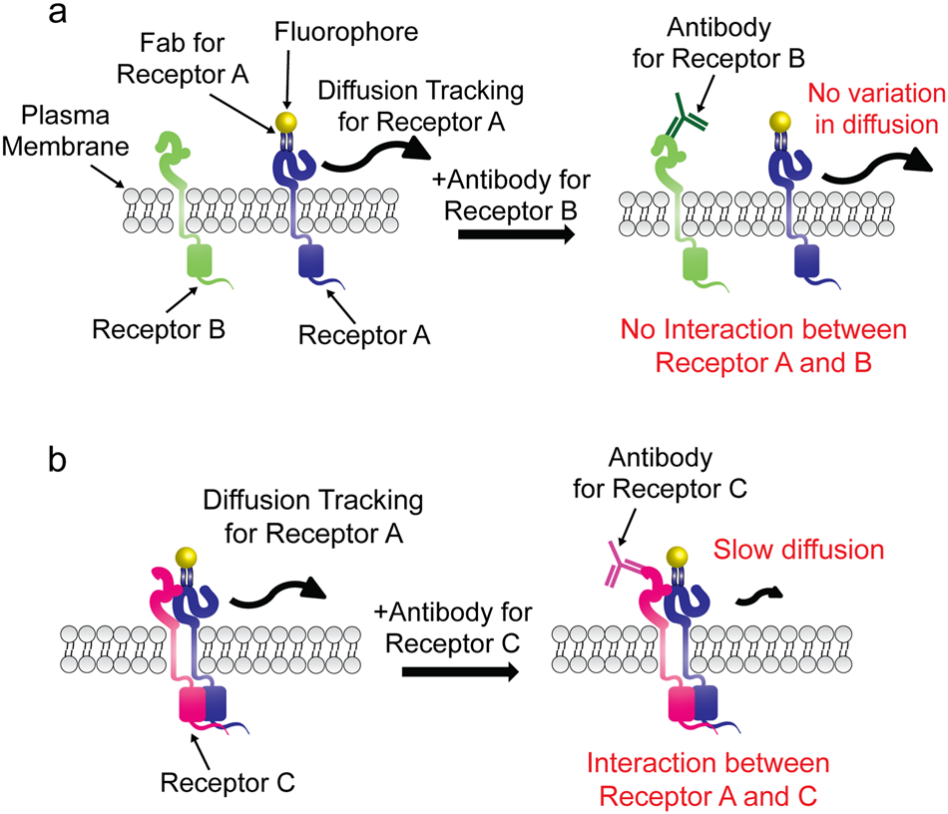
Schematic representation of the modified single-molecule diffusional mobility shift assay (smDIMSA) used for interaction analysis. **a** Diffusion of receptor A is not disrupted by receptor B even with antibody treatment because receptor B is a noninteracting target of receptor A. **b** When receptor A interacts with receptor C, the antibody targeting receptor C causes a shift in the diffusion coefficient.

Recently, we revealed that the diffusion coefficient of membrane proteins varies when soluble ligands bind to membrane proteins, which could not be expected according to the Saffman–Delbrück model, a previous standard model explaining the diffusion of proteins on a membrane. This method can detect the variations in the diffusion coefficient of membrane proteins when soluble ligands bind to membrane proteins and can be sensitively and robustly detected by the spatio-temporal average of single-molecule diffusivity obtained using single-particle tracking with super-resolution microscopy, called smDIMSA^19^. We revised the smDIMSA technique to measure transient interactions on the plasma membrane of living cells by establishing a prey-bait system. When the prey and bait proteins interact, their diffusivity is shared because they physically form a complex. The diffusion coefficient of the bait will be reduced when the specific antibody targeting the prey is applied only if the prey and the bait form a complex; otherwise, the diffusion coefficient of the bait is unperturbed (Fig. 1). The practical implementation of this intuitive idea was very simple; the individual bait proteins are visualized in a living cell by super-resolution microscopy before and after the addition of the anti-prey antibody.

### Validation of smDIMSA for identification of interactions using CD86 and CD28

To validate the capability of interaction identification using smDIMSA, we used a previously reported constitutive plasma membrane monomer and dimer, namely, CD86 and CD28, respectively^23,24^. Because we aimed to measure interactions with endogenous proteins, we tagged CD86 and CD28 with fluorophores extrinsically rather than genetically. In this experiment, we cannot differentiate between the prey and bait—the two are identical, since we need to measure homodimers of CD86 and CD28. Thus, we modified our strategy to exploit competition by using the same antibody to label the bait protein and shifting the diffusion coefficient of the prey protein. Monoclonal antibodies were used because nonspecific clustering can be induced by polyclonal antibodies, which bind to multiple epitopes of the target protein. We also selected functionally neutral antibodies to minimize the possible interference of antibody binding on the protein–protein interaction^9^. Then, we generated Alexa Fluor 647-conjugated Fab fragments of each antibody specific for CD86 and CD28 to partially label a small fraction of the bait proteins, which is sufficient to obtain the diffusion coefficient of the single-molecule bait. This strategy guarantees that the anti-prey antibody does not bind to the visualized bait; since the fluorescently labeled Fab fragment and the nonlabeled anti-bait antibody share the epitope, either the Fab fragment or the full antibody can bind to one protein simultaneously. Since CD86 is a constitutive monomer, we expected that the anti-prey antibody would not be able to bind because the binding epitope of CD86 was occupied by the anti-bait-targeted Fab fragment. However, CD28 is a constitutive dimer, and it has an open binding epitope on the dimerization partner, even when the Fab fragment occupies the binding epitope on the bait protein. Therefore, if a homodimer of a Fab-occupied and an antibody-bound receptor is formed, we should be able to detect a shift in the diffusion coefficient distribution of the Fab-labeled receptor and vice versa. The extent of the diffusion shift is an indicator of the ratio of pre-homodimer formation. Although the labeling ratio should influence the diffusion shift detected, this influence can be overcome if the Fab concentration is kept constant during measurement, according to binding theory.

**Fig. 2:**
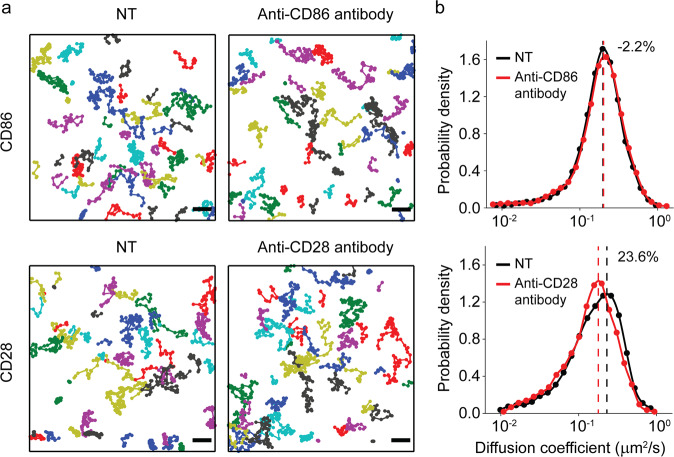
Antibody-mediated shift in CD86 and CD28. **a** Trajectory maps of CD86–mEos3.2 and CD28–mEos3.2 in HeLa cells before and after antibody treatment. Fifty trajectories are shown in each trajectory map. Scale bars, 1 μm. **b** CD86–mEos3.2 and CD28–mEos3.2 before (black line) and after antibody treatment (red line). The dashed lines indicate the peak of each colored line.

We expressed CD86 and CD28 in HeLa cells (Fig. 2). To minimize interference from the cytoplasmic domain, the intracellular regions of both proteins were truncated^20^. The monomeric fluorescent tag mEos3.2 was added at the N-terminus as an expression reporter. We first used smDIMSA to prove whether antibody binding induces a diffusion shift of CD86 and CD28 by tracking individual molecules of mEos3.2.

Then, to detect homodimerization, we used CD28–mEos3.2 and CD86–mEos3.2, which were later labeled with anti-CD28 Fab-Alexa Fluor 647 and anti-CD86 Fab-Alexa Fluor 647, respectively. CD28 and CD86 were both treated with the full-length antibody after labeling with the fluorescent dye to visualize homodimerization. Since the full-length antibody and Fab fragment share a binding epitope, if a protein does not dimerize, the diffusion coefficient is not changed.

We partially labeled CD28 and CD86 with the Alexa Fluor 647-conjugated Fab fragment. The diffusion coefficient distribution of the Fab-labeled CD86 protein was unaffected by treatment with either the full-length anti-CD86 antibody or nonspecific IgG (Fig. 2b and Supplementary Fig. 3a). Although IgG treatment did not cause any changes (Supplementary Fig. 3a), full-length anti-CD28 antibody treatment induced a shift, indicating that the CD28 homodimer was detected (Fig. 2a, b). The peak shift averaged from multiple cells was ~18% for CD28 and <1% for CD86 (Supplementary Fig. 3b), which proves the applicability of the modified smDIMSA to receptors other than EGFR.

### Validation of smDIMSA for analysis of EGFR interactions

**Fig. 3:**
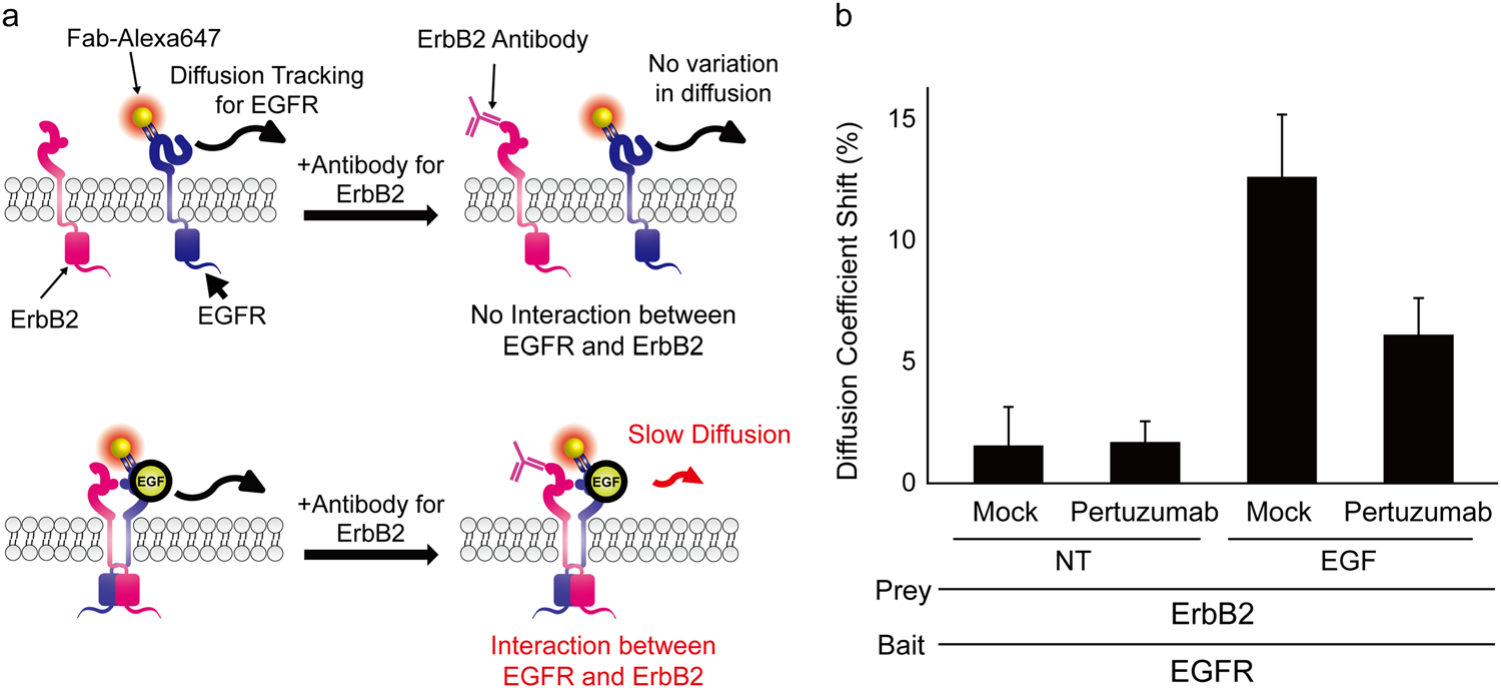
The interaction between EGFR and ErbB2 only exists under EGF-treated conditions in noncancer cell lines. **a** Schematic for visualizing the EGFR–ErbB2 interaction. Under the EGF-nontreated condition, EGFR and ErbB2 do not interact (upper panel), but interaction occurs when EGF is present (lower panel). **b** ErbB2 does not interact with EGFR under the EGF-nontreated (NT) condition. When EGF is present, ErbB2 interacts with EGFR, and application of an antibody targeting ErbB2 causes a shift in the EGFR diffusion coefficient, which is then disrupted by treatment with pertuzumab, an antibody drug that blocks ErbB2 heterodimerization. All error bars indicate the standard error of the mean (s.e.m.) values for a single-cell population (*n* ≥ 5).

Prior to proceeding with smDIMSA, validation of the modified smDIMSA with EGFR was required, and this verification was performed for the interaction between EGFR and ErbB2, a member of the ErbB family of receptors. EGFR heterodimerizes with all ErbB family members, and we used the EGFR–ErbB2 pair to confirm the validity of the modified smDIMSA. We used EGFR as the bait protein and ErbB2 as the prey protein. Fab-Alexa Fluor 647-tagged EGFR was observed over time to acquire its trajectories, and cells were then treated with an anti-ErbB2 antibody to determine whether EGFR and ErbB2 interact by observing whether the diffusion coefficient changes under EGF-treated or nontreated condition (Fig. 3a).

To visualize the EGFR–ErbB2 interaction status, we used pertuzumab, an anticancer drug that binds to domain II of ErbB2 to block its dimerization with ErbB family members. We first evaluated the EGFR–ErbB2 interaction in Cos7 cells without ligand treatment and saw a minimal shift (~1.5%) in the diffusion coefficient (Fig. 3b). However, treatment with pertuzumab under this condition also had a minimal effect, proving that EGFR and ErbB2 do not interact in Cos7 cells not treated with ligand (NT). Under EGF treatment conditions, however, the mock cells exhibited a shift in the diffusion coefficient of ~12% (Fig. 3b), showing that smDIMSA detected a shift in the movement of EGFR when the ErbB2 antibody was present, thereby indicating an interaction between the two proteins. This interaction was perturbed in the presence of pertuzumab, which lowered the shift to ~6%. Thus, modified smDIMSA can detect a shift in the diffusion coefficient when the prey protein and bait protein interact and the antibody targeting the prey protein is present by increasing the hydrodynamic radius, thereby validating the method’s applicability in measuring the interaction between two receptors.

### Promiscuity of EGFR interactions in various cell lines

**Fig. 4:**
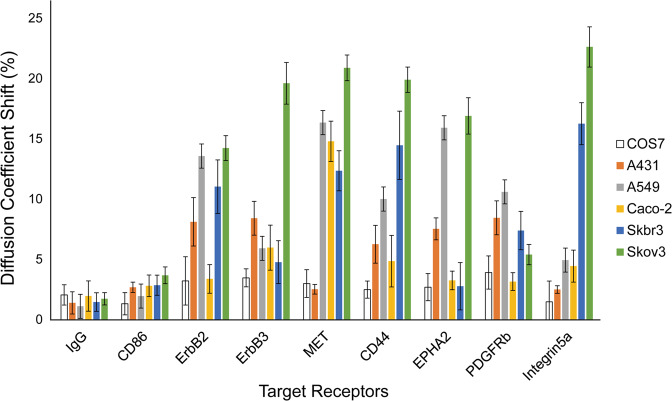
The interaction pattern of EGFR with different receptors in different cell lines. EGFR was used as the bait and each receptor was used as prey in the presence of an antibody targeting the prey. Diffusion coefficient shifts were measured for all receptors in cancer cell lines (A431, A549, NCI-H508, Caco-2, Skbr3, and Skov3) and Cos7 cells. All error bars indicate the standard error of the mean (s.e.m.) values for a single-cell population (*n* ≥ 5).

We analyzed the interactions between EGFR and all RTKs reported to interact with EGFR–ErbB2, ErbB3, MET, CD44, EPHA2, PDGFRb, and integrin5a—in the cell lines. These receptors were selected because they interact with EGFR in cancer cells. ErbB2 and ErbB3 interact with EGFR in cancer cells^25^; MET receptors, CD44, EPHA2, and PDGFRb are implicated in resistance to EGFR-targeted drugs by circumventing the signaling pathway that the drug targets^26–29^. Integrin5a also interacts with EGFR in cancer cells^12^. Moreover, when visualizing receptors, antibody treatment should not cause additional effects; thus, using a neutral antibody is a prerequisite for the experiments. We decided to broaden the spectrum of our study by observing the interaction between EGFR and other receptors in various cell lines. Since our bait protein was EGFR, we decided to use cetuximab-resistant and cetuximab-sensitive cell lines. We selected Skbr3^30^ and Skov3^31^ as cetuximab-resistant cell lines and A431^32^, A549^33^, and Caco-2 as cetuximab-sensitive cell lines; these cell lines express varied levels of EGFR. A431 has relatively high EGFR expression levels^34^, as does Cos7, whereas A549, Skbr3, Skov3, and Caco-2^35,36^ have moderate-to-low EGFR expression levels. However, A431 has the lowest average diffusion coefficient shift among cancer cell lines, whereas Skbr3 and Skov3 have the highest average shifts, indicating the reason that the expression level of EGFR does not thoroughly control its activation^37^. We show the diffusion coefficient distribution plot for all receptors in different cell lines to support our data in Fig. 4 (Supplementary Figs. 4–9).

All cancer cell lines except Caco-2 (3.4%) showed a high interaction level with ErbB2, as ErbB2 is another major interacting partner of EGFR in addition to EGFR itself. However, ErbB3 only showed a high diffusion coefficient shift in Skov3 cells (19.6%). All cell lines showed distinct interaction patterns with prey receptors, and MET, CD44, and integrin5a showed the highest average shifts in cancer cell lines—13.38%, 11.10%, and 10.15%, respectively. The cell lines with the most unique features were Caco-2 and Skov3. Caco-2 cells exhibited minimal interactions of EGFR with most receptors except MET (14.8%), although ErbB2 and ErbB3 are the main interacting partners of EGFR (Fig. 4). This is an interesting finding because previously published data indicated that EGFR and MET interact with each other in the Caco-2 cell line, as shown by Co-IP^38^. Similar results were obtained for ErbB2, which was shown to interact with EGFR in the Skbr3 and Skov3 cell lines^39^; our data are consistent with that data, showing shifts of 11.03% and 14.23%, respectively, in these cell lines. The interaction between EGFR and EphA2 was also observed using Co-IP in the A431 cell line^40^, consistent with our data, which showed a shift of 10.05% for the interaction between EGFR and EphA2 in the A431 cell line. Although there are many studies that evaluated the interactions between EGFR and the other RTKs that we selected, such as CD44^41^, MET^42^, and EphA2^28^, these studies were not performed in the same cell lines, as Co-IP for these interaction pairs was performed in MDA-MB-231, H1975, and HEK293T cell lines. However, the interaction between EGFR and many other RTKs occurs to varying degrees, as presented by our data.

## Discussion

We demonstrated that smDIMSA can be used as an analytical tool for evaluating transient protein–protein interactions in the plasma membrane of living cells at the single-molecule level. Although live-cell protein–protein interaction measurement methods, such as FRET and BRET, exist, smDIMSA can overcome the limitations of these methods, such as fluorophore orientation problems and the need for additional labeling to visualize interactions. Moreover, problems of proximity-based methods, including the false-positive rate governed by crowdedness, are easily solved through diffusion-based methods, as diffusion coefficient shifts only occur when the two receptors interact. With its properties such as single-cell sensitivity, molecular specificity, and lack of a need for ligand-non-labeling property, smDIMSA can greatly facilitate target-based drug development or drug assessment. Since smDIMSA measures interactions between ligand and membrane proteins in live cells without the need for purification of the target protein, both false positives and false negatives resulting from the use of purified membrane proteins with an incomplete structure due to the lack of a lipid membrane or other factors may be reduced. In this study, by simply changing the cell lines and target receptors, we proved that the interaction profiles are quite distinct among cell lines and that the expression level of a receptor does not govern the interaction level. With minimal disruption of the cell, this method allows the acquisition of an interaction profile at the single-cell level, which is an important feature, especially in the medical field. Medical analysis still primarily uses expression analysis or recurrence evaluation approaches for diagnosis^43^, which do not thoroughly explain or predict EGFR’s behavior^44^, as interactomics does. Our method is experimentally tractable and sensitive, and it can be applied to pioneer interactomics studies in the medical field.

Although the expression profile has historically been thought to dominate the interaction profile, previous reports indicate that a high expression level does not solely determine the activation status of a receptor^44^. As seen in our data, all cell lines showed different interaction behaviors, which were not dependent on the EGFR expression level. Interestingly, the Skbr3 and Skov3 cell lines, which are cetuximab-resistant, showed a high diffusion coefficient shift for MET, CD44, and integrin5a. Moreover, the Skov3 cell line showed a high diffusion coefficient shift even for ErbB2 and ErbB3, suggesting that EGFR can circumvent the inhibition of EGFR pathway activation via many mechanisms. Notably, although A431 cells expressed the highest level of EGFR, they exhibited the lowest interaction of EGFR with its prey receptors of all cells except Cos7 cells, which are not of human origin and are noncancerous. By applying smDIMSA, we may understand the etiology of resistance to targeted drug therapies.

While mutations or alterations in downstream effectors are considered the pertinent mechanisms of drug resistance, interactions between receptors, or heterointeractions, are also known causes of resistance^45^. Since heterointeractions between EGFR and other RTKs play an essential role in cancers, many papers have tried to decode these heterointeractions. Interactions between EGFR and CD44, MET and the ErbB2 receptor have been observed using Co-IP^39,41,42,46^, while EGFR and EphA2 have been shown to interact by Co-IP^40^ and FRET^28^. Some studies have even indicated that heterointeraction between EphA2 and EGFR is the cause of poor prognosis and poor response to cetuximab due to overexpression in colorectal cancer^28,47^. In addition, MET is a known favorable interacting partner in cancer, and evidence indicates that MET induces resistance to anti-EGFR drugs in NSCLC^28^. Our data are consistent with those of other studies^48^, and we show the level of interaction within the plasma membrane. Membrane protein interactions are transient in nature, and the interactions that can be visualized using non-live-cell methods such as Co-IP indicate that interactions between plasma membrane proteins can be highly abundant. However, smDIMSA is highly sensitive for analyzing interactions and can detect even very low levels of interactions between receptors.

Although the diffusion coefficient was changed by antibody binding for many receptors and antibodies we examined, optimization steps are required before smDIMSA can be applied. First, neutral antibodies targeting the extracellular domain must exist. Such antibodies against membrane proteins that have been extensively investigated can be obtained readily, but this might not be the case for many membrane proteins, including G protein-coupled receptors. Second, the saturating concentration of the antibody should be determined prior to applying this method, because the dissociation constant varies among antibodies and target proteins. To avoid possible dimerization induced by the bivalency of an antibody, the antibody should be applied at its saturating concentration so that the target receptors are occupied mostly by excess free antibody in solution before the antibody bound to the receptor diffuses to contact another free receptor. Third, the diffusion coefficient shift induced by antibody binding should be verified. The main concept of this method is that membrane proteins exist primarily as monomers and are constantly mobile on the plasma membrane. Although this condition is satisfactory for analyzing RTK dimerization, it may not be appropriate for analyzing membrane proteins related to structural scaffolds.

## Supplementary information

Supplementary Figures and a Table

## References

[1] Jennings ML (1989). Topography of membrane proteins. Annu. Rev. Biochem..

[2] Babu M (2012). Interaction landscape of membrane-protein complexes in Saccharomyces cerevisiae. Nature.

[3] Phillips R, Ursell T, Wiggins P, Sens P (2009). Emerging roles for lipids in shaping membrane-protein function. Nature.

[4] Leschziner AE, Nogales E (2007). Visualizing flexibility at molecular resolution: analysis of heterogeneity in single-particle electron microscopy reconstructions. Annu. Rev. Biophys..

[5] Solomatin SV, Greenfeld M, Herschlag D (2011). Implications of molecular heterogeneity for the cooperativity of biological macromolecules. Nat. Struct. Mol. Biol..

[6] Wisniewski JR, Zougman A, Nagaraj N, Mann M (2009). Universal sample preparation method for proteome analysis. Nat. Methods.

[7] Miernyk JA, Thelen JJ (2008). Biochemical approaches for discovering protein-protein interactions. Plant J..

[8] Kerppola TK (2008). Bimolecular fluorescence complementation (BiFC) analysis as a probe of protein interactions in living cells. Annu Rev. Biophys..

[9] Villalobos V, Naik S, Piwnica-Worms D (2007). Current state of imaging protein-protein interactions in vivo with genetically encoded reporters. Annu. Rev. Biomed. Eng..

[10] Lippincott-Schwartz J, Snapp E, Kenworthy A (2001). Studying protein dynamics in living cells. Nat. Rev. Mol. Cell Biol..

[11] Sprague BL, McNally JG (2005). FRAP analysis of binding: proper and fitting. Trends Cell Biol..

[12] Kuninty PR (2019). ITGA5 inhibition in pancreatic stellate cells attenuates desmoplasia and potentiates efficacy of chemotherapy in pancreatic cancer. Sci. Adv..

[13] Betzig E (2006). Imaging intracellular fluorescent proteins at nanometer resolution. Science.

[14] Rust MJ, Bates M, Zhuang X (2006). Sub-diffraction-limit imaging by stochastic optical reconstruction microscopy (STORM). Nat. Methods.

[15] Jungmann R (2014). Multiplexed 3D cellular super-resolution imaging with DNA-PAINT and exchange-PAINT. Nat. Methods.

[16] Kanchanawong P (2010). Nanoscale architecture of integrin-based cell adhesions. Nature.

[17] Sengupta P (2011). Probing protein heterogeneity in the plasma membrane using PALM and pair correlation analysis. Nat. Methods.

[18] Strauss S, Jungmann R (2020). Up to 100-fold speed-up and multiplexing in optimized DNA-PAINT. Nat. Methods.

[19] Kim DH (2015). Analysis of Interactions between the epidermal growth factor receptor and soluble ligands on the basis of single-molecule diffusivity in the membrane of living cells. Angew. Chem. Int. Ed. Engl..

[20] Dorsch S, Klotz KN, Engelhardt S, Lohse MJ, Bunemann M (2009). Analysis of receptor oligomerization by FRAP microscopy. Nat. Methods.

[21] Kim DH (2017). Single particle tracking-based reaction progress kinetic analysis reveals a series of molecular mechanisms of cetuximab-induced EGFR processes in a single living cell. Chem. Sci..

[22] Jaqaman K (2008). Robust single-particle tracking in live-cell time-lapse sequences. Nat. Methods.

[23] Bhatia S, Edidin M, Almo SC, Nathenson SG (2005). Different cell surface oligomeric states of B7-1 and B7-2: implications for signaling. Proc. Natl Acad. Sci. USA..

[24] Lazar-Molnar E, Almo SC, Nathenson SG (2006). The interchain disulfide linkage is not a prerequisite but enhances CD28 costimulatory function. Cell. Immunol..

[25] Ma J, Lyu H, Huang J, Liu B (2014). Targeting of erbB3 receptor to overcome resistance in cancer treatment. Mol. Cancer.

[26] Jo M (2000). Cross-talk between epidermal growth factor receptor and c-Met signal pathways in transformed cells. J. Biol. Chem..

[27] Perez A (2013). CD44 interacts with EGFR and promotes head and neck squamous cell carcinoma initiation and progression. Oral. Oncol..

[28] Paul MD, Grubb HN, Hristova K (2020). Quantifying the strength of heterointeractions among receptor tyrosine kinases from different subfamilies: implications for cell signaling. J. Biol. Chem..

[29] Black PC (2011). Receptor heterodimerization: a new mechanism for platelet-derived growth factor induced resistance to anti-epidermal growth factor receptor therapy for bladder cancer. J. Urol..

[30] Uberall I, Krizova K, Steigerova J (2011). Cetuximab enhances the anti-proliferative effect of trastuzumab in ERBB2 over-expressing breast cancer cells-preliminary study. Klin. Onkol..

[31] Gottschalk N, Kimmig R, Lang S, Singh M, Brandau S (2012). Anti-epidermal growth factor receptor (EGFR) antibodies overcome resistance of ovarian cancer cells to targeted therapy and natural cytotoxicity. Int. J. Mol. Sci..

[32] Yang X (2013). Cetuximab-mediated tumor regression depends on innate and adaptive immune responses. Mol. Ther..

[33] Hsu YF (2010). Complement activation mediates cetuximab inhibition of non-small cell lung cancer tumor growth in vivo. Mol. Cancer.

[34] Bjorkelund H, Gedda L, Barta P, Malmqvist M, Andersson K (2011). Gefitinib induces epidermal growth factor receptor dimers which alters the interaction characteristics with (1)(2)(5)I-EGF. PLoS ONE.

[35] Sun R (2015). Cloning and expression of a novel target fusion protein and its application in anti-tumor therapy. Cell. Physiol. Biochem..

[36] Yu X (2018). Targeting EGFR/HER2/HER3 with a three-in-one aptamer-siRNA chimera confers superior activity against HER2(+) breast cancer. Mol. Ther. Nucleic Acids.

[37] Nijkamp MM, Span PN, Bussink J, Kaanders JH (2013). Interaction of EGFR with the tumour microenvironment: implications for radiation treatment. Radiother. Oncol..

[38] Song N (2014). Cetuximab-induced MET activation acts as a novel resistance mechanism in colon cancer cells. Int. J. Mol. Sci..

[39] DeFazio-Eli L (2011). Quantitative assays for the measurement of HER1-HER2 heterodimerization and phosphorylation in cell lines and breast tumors: applications for diagnostics and targeted drug mechanism of action. Breast Cancer Res.

[40] Larsen AB (2007). Activation of the EGFR gene target EphA2 inhibits epidermal growth factor-induced cancer cell motility. Mol. Cancer Res..

[41] Grass GD, Tolliver LB, Bratoeva M, Toole BP (2013). CD147, CD44, and the epidermal growth factor receptor (EGFR) signaling pathway cooperate to regulate breast epithelial cell invasiveness. J. Biol. Chem..

[42] Ortiz-Zapater E (2017). MET-EGFR dimerization in lung adenocarcinoma is dependent on EGFR mtations and altered by MET kinase inhibition. PLoS ONE.

[43] Wang Q (2019). Gene expression profiling for diagnosis of triple-negative breast cancer: a multicenter, retrospective cohort study. Front. Oncol..

[44] Kuwada SK (2004). Effects of trastuzumab on epidermal growth factor receptor-dependent and -independent human colon cancer cells. Int. J. Cancer.

[45] Paul MD, Hristova K (2019). The RTK interactome: overview and perspective on RTK heterointeractions. Chem. Rev..

[46] Wang SJ, Bourguignon LY (2006). Hyaluronan and the interaction between CD44 and epidermal growth factor receptor in oncogenic signaling and chemotherapy resistance in head and neck cancer. Arch. Otolaryngol. Head. Neck Surg..

[47] De Robertis M (2017). Dysregulation of EGFR pathway in EphA2 cell subpopulation significantly associates with poor prognosis in colorectal cancer. Clin. Cancer Res..

[48] Reinmuth N (2009). Combined anti-PDGFRalpha and PDGFRbeta targeting in non-small cell lung cancer. Int J. Cancer.

