# Supplementary material for: Analysis of transient membrane protein interactions by single-molecule diffusional mobility shift assay
Source: Exp Mol Med. 2021 Feb 19;53(2):291–9. doi: 10.1038/s12276-021-00567-1 (PMC8080847; doi:10.1038/s12276-021-00567-1)
Supplement: Supplementary file 1 — Supplementary Figures and a Table [file 12276_2021_567_MOESM1_ESM.docx]

**Supplementary Figures and Table**


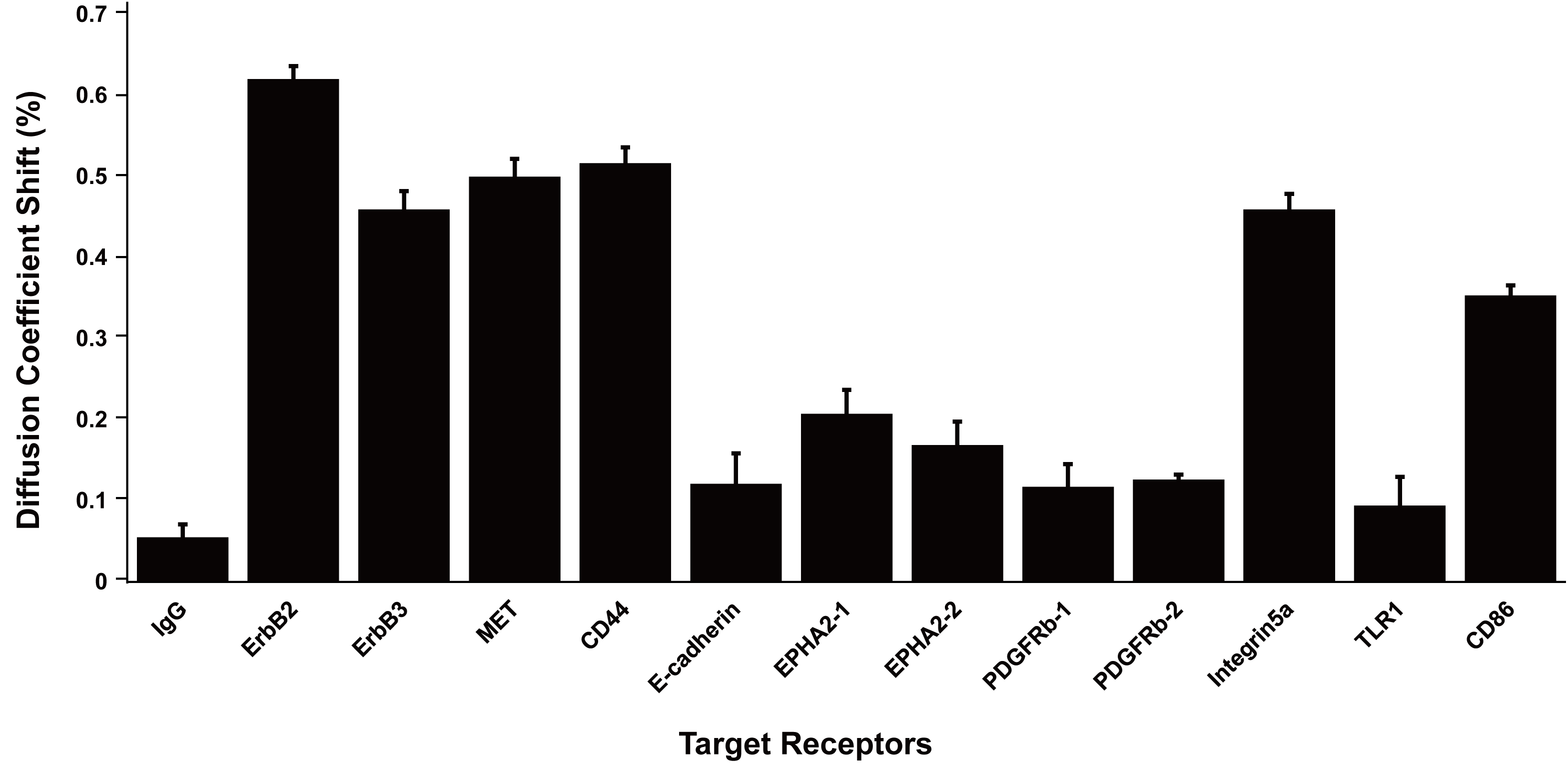


Supplementary Figure 1. Antibody mediated diffusion coefficient shift in different receptors. Instead of utilizing the bait-prey interaction pair, each receptor is used as a prey, targeted by antibodies while being observed simultaneously with mEos3.2 fluorescent protein which is genetically tagged to each receptors. EPHA2 and PDGFRb have two different kinds of antibodies, and each antibody showed a similar shift in diffusion coefficient. All receptors showed a significant amount of diffusion coefficient shift value compared to IgG. All error bars indicate standard error of mean (s.e.m.) of a single-cell population (n≥5).


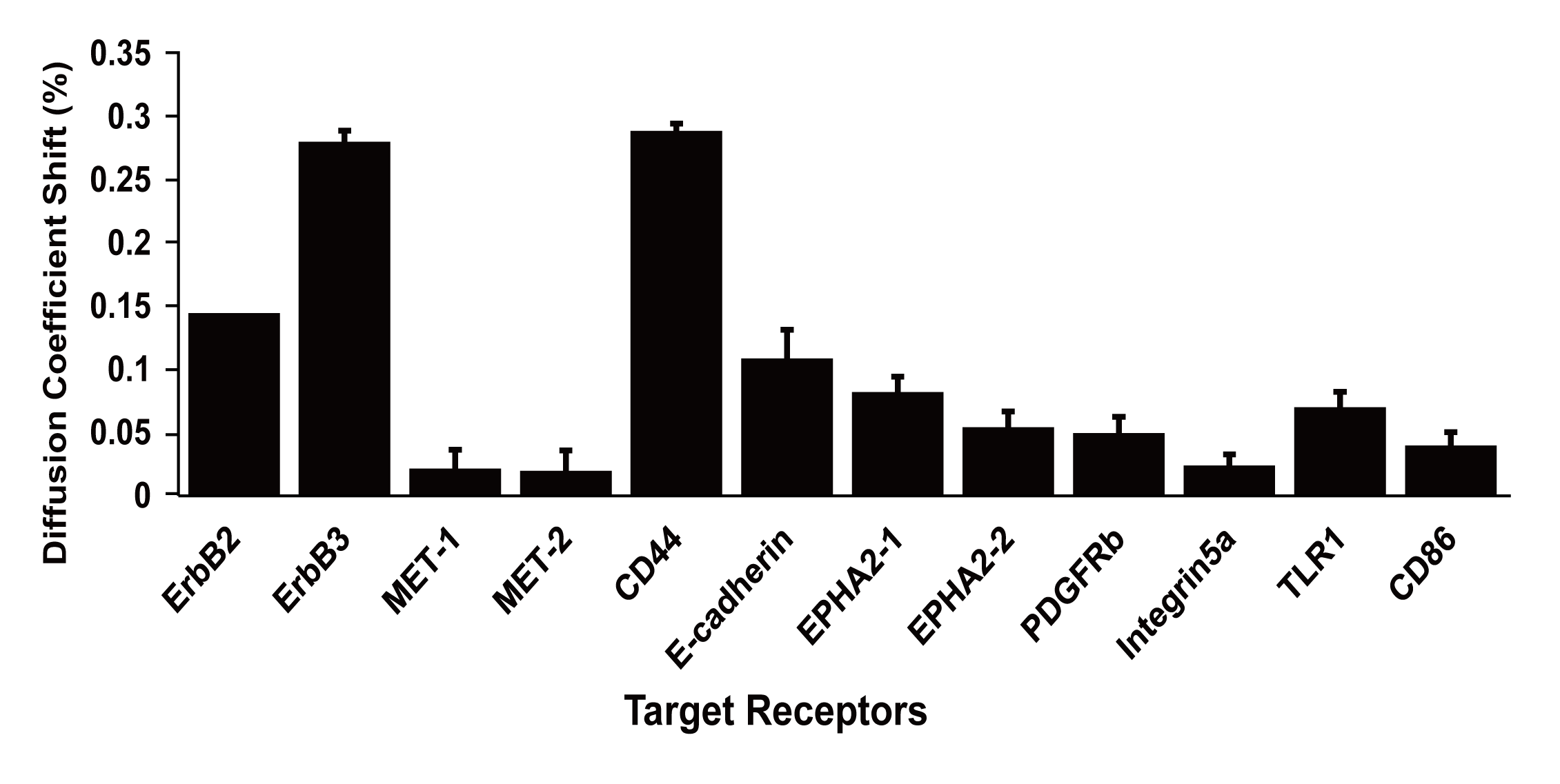


Supplementary Figure 2. Diffusion coefficient shift from the target receptor and EGFR interaction. Utilizing the bait-prey interaction pair where EGFR is the bait protein tagged with Fab–Alexa Fluor 647. Antibody targeting each of the prey receptors are treated to visualize shift in diffusion coefficient of EGFR, the bait protein. All error bars indicate s.e.m. of a single-cell population (n≥5).


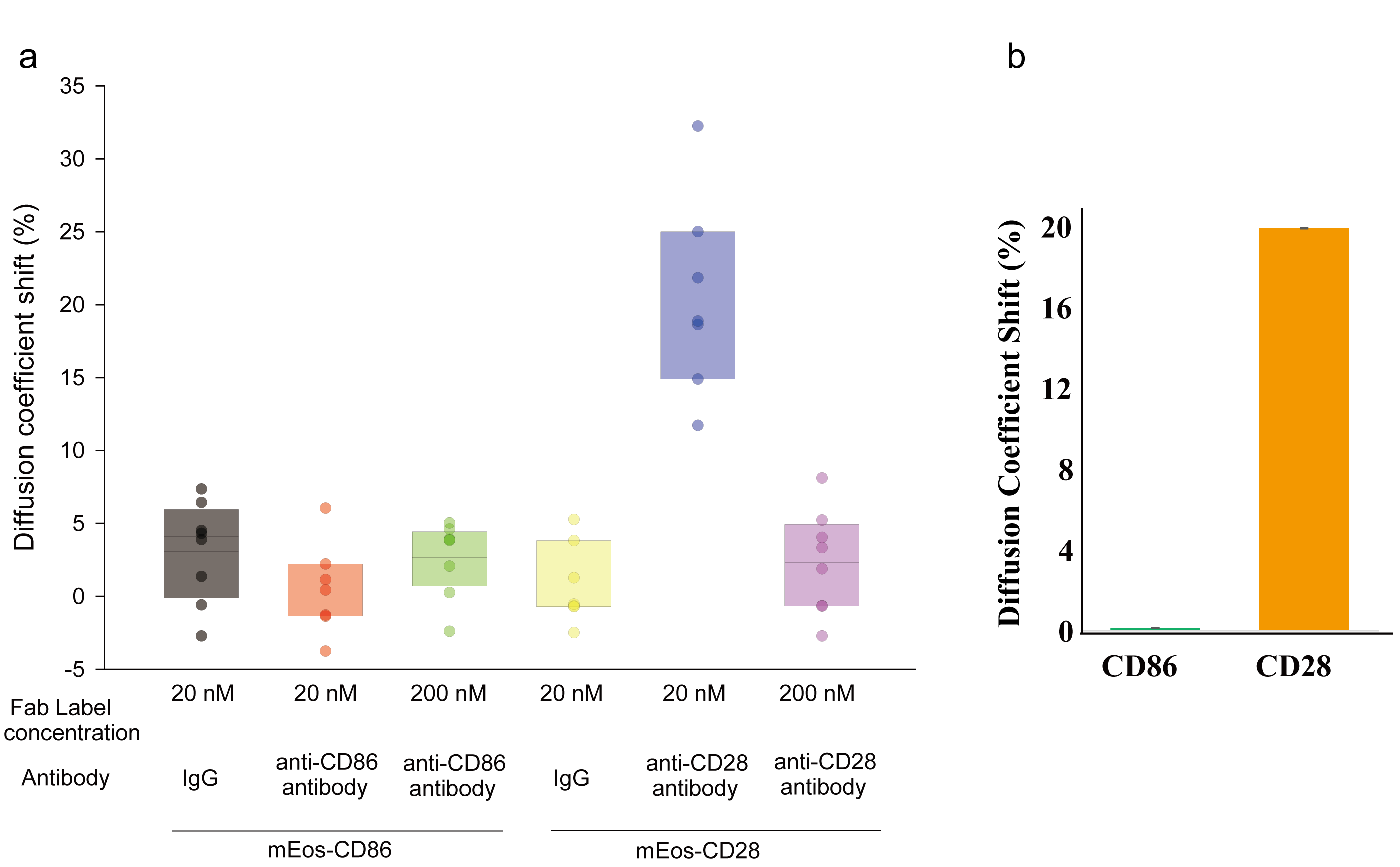


Supplementary Figure 3. Partial labeling and saturation labeling shows different result in CD28 and CD86. a CD28 is a homodimer, so partial labeling would leave a single epitope for a full-length antibody to bind, thus leading to diffusion coefficient shift. However, CD86 is a monomer and Fab bound receptor would not have full-length antibody bind to it, because the full-length antibody’s binding epitope is already occupied by Fab, resulting in no shift in diffusion coefficient. This is possible with partial labeling, but when saturating concentration of the Fab is treated, even CD28 does not have any binding epitope available, thus leading to no shift in diffusion coefficient even after the full-length antibody is treated. b Diffusion coefficient shift of CD86 and CD28. Averaged diffusion coefficient shift of CD86 was 0.13% and diffusion coefficient shift of CD28 was 19.9%


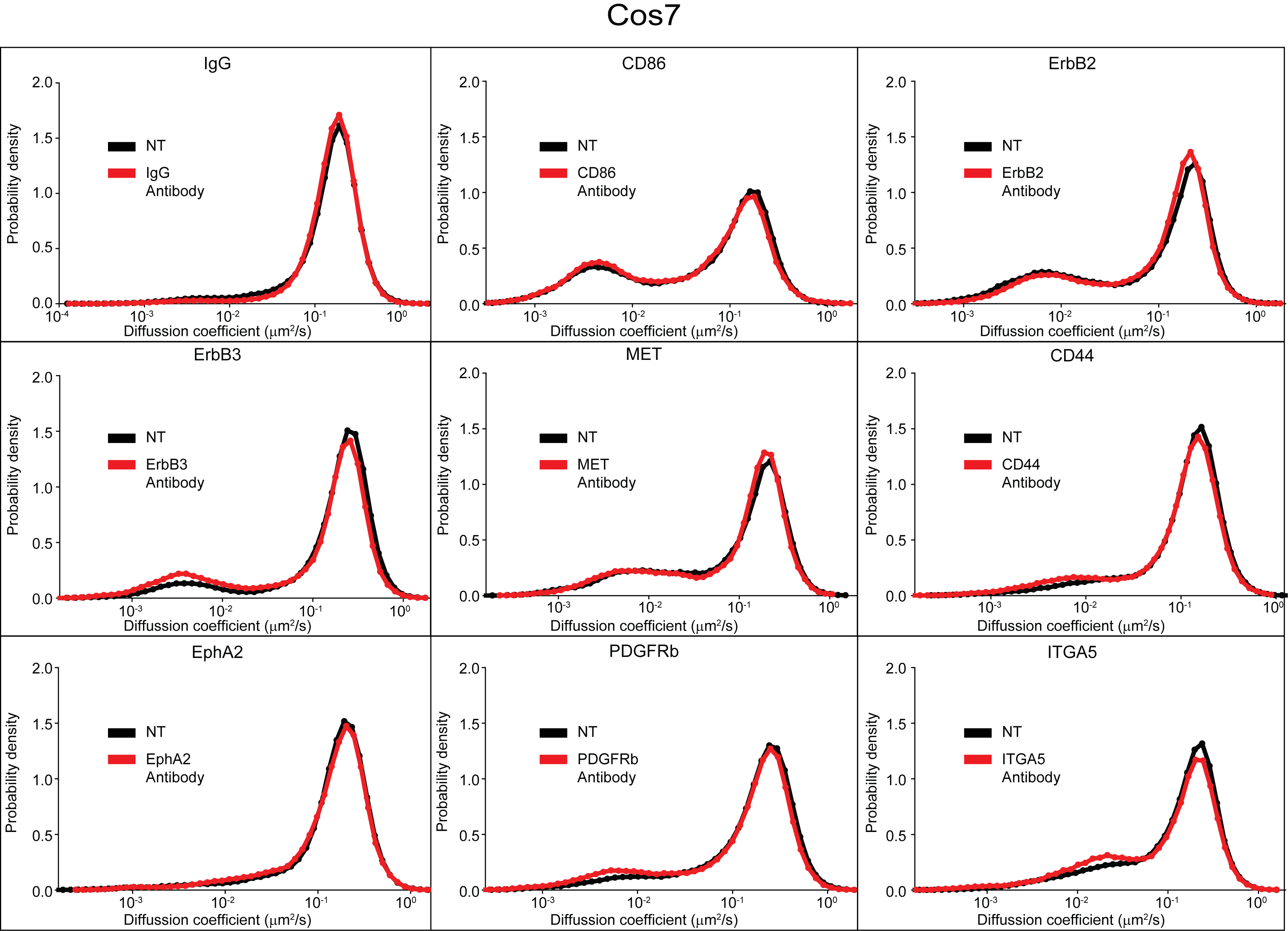


Supplementary Figure 4. Diffusion coefficient distribution for Cos7 cell. Diffusion coefficient distribution histogram for all targets. Black line indicates population distribution before antibody treatment and red line indicates the population distribution after the antibody was treated. The shift in diffusion coefficient is as follows. IgG: 2.10%, CD86: 3.13%, ErbB2: 4.94%, ErbB3: 4.59%, MET: 1.98%, CD44: 2.88%, EphA2: 2.52%, PDGFRb: 3.11%, Integrin5a: 1.12%.


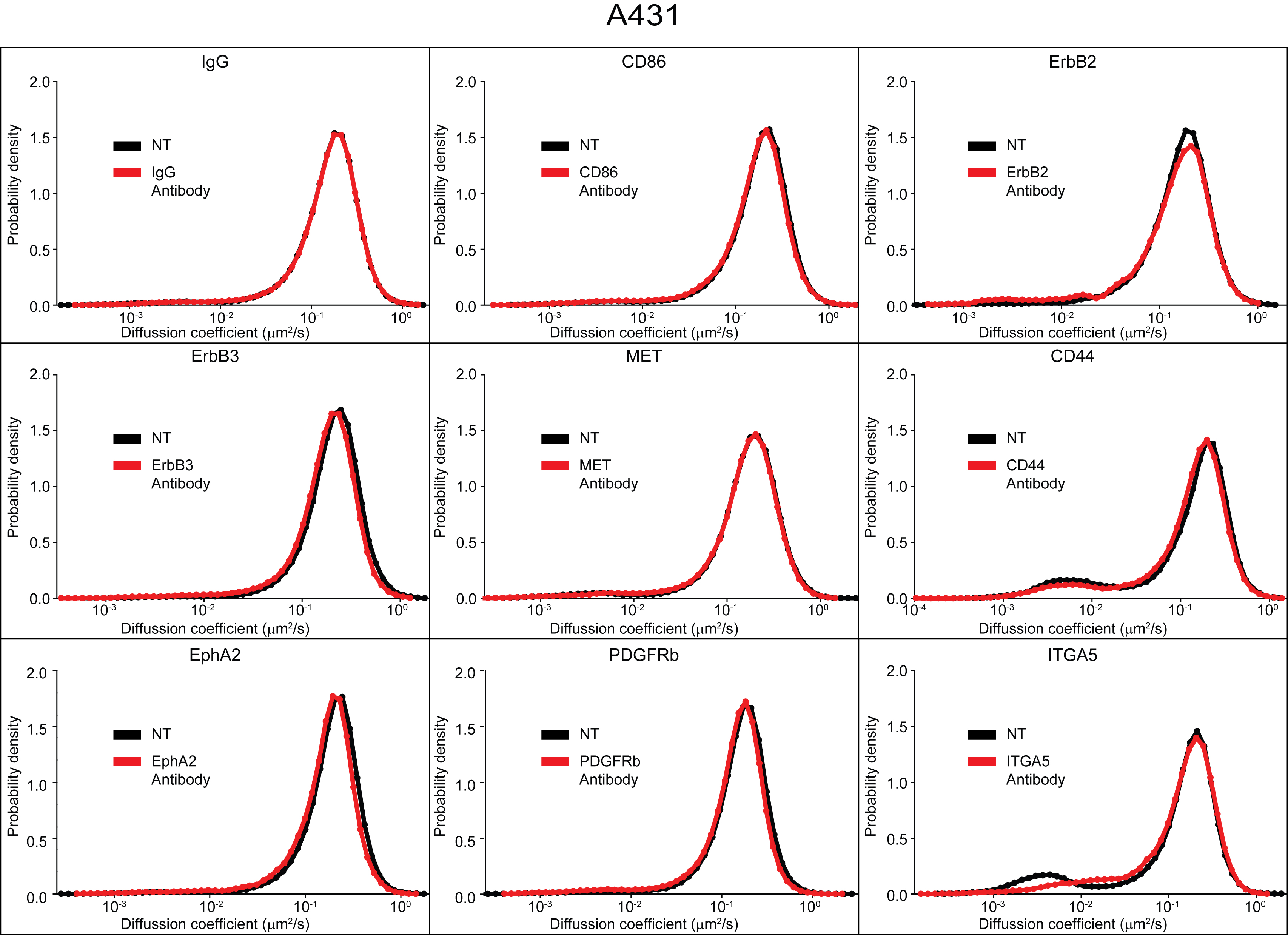


Supplementary Figure 5. Diffusion coefficient distribution for A431 cell. Diffusion coefficient distribution histogram for all targets. Black line indicates population distribution before antibody treatment and red line indicates the population distribution after the antibody was treated. The shift in diffusion coefficient is as follows. IgG: -0.23%, CD86: 3.59%, ErbB2: 9.13%, ErbB3: 10.03%, MET: 2.01%, CD44: 7.66%, EphA2: 10.05%, PDGFRb: 7.79%, Integrin5a: 3.44%.


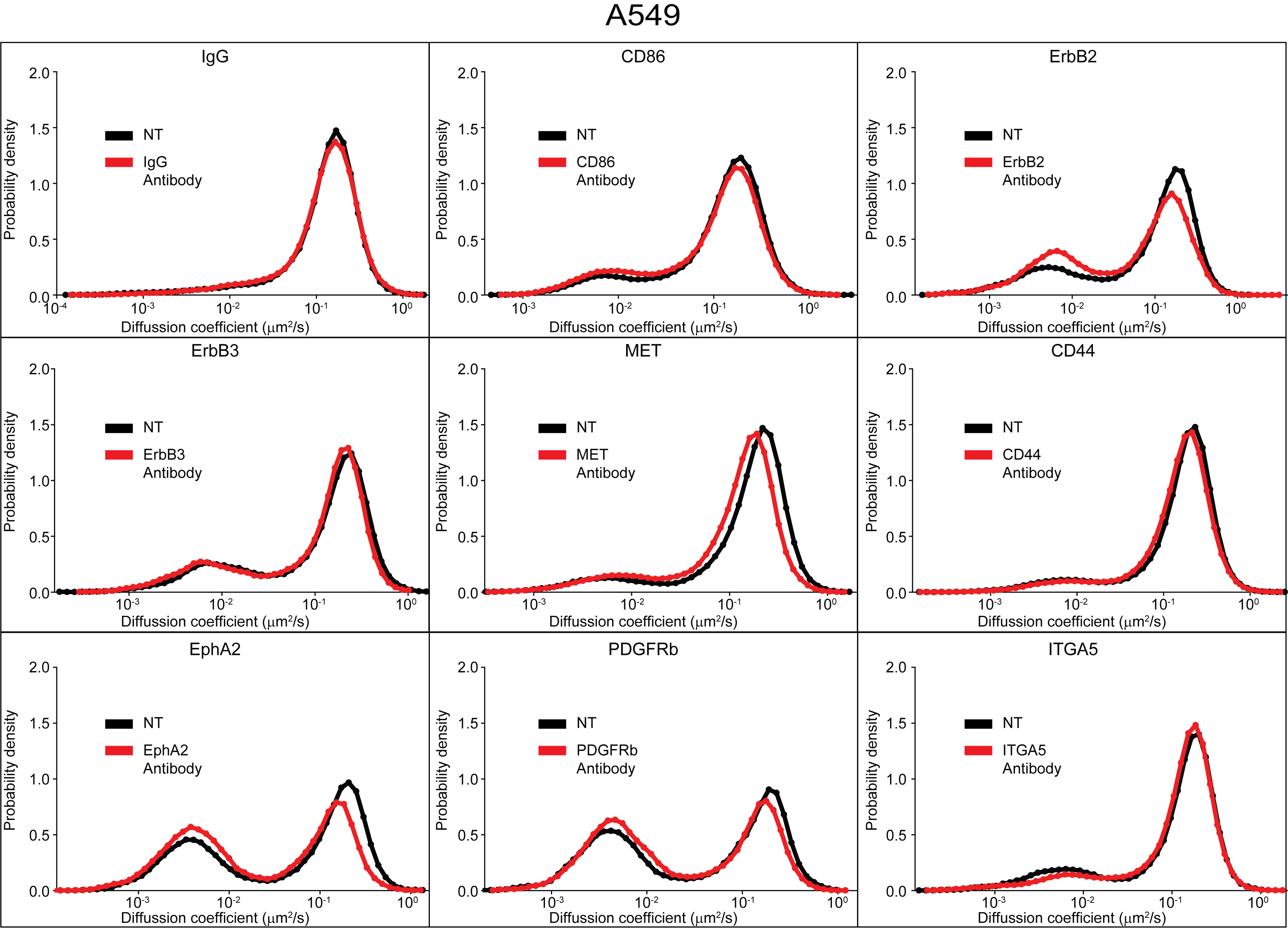


Supplementary Figure 6. Diffusion coefficient distribution for A549 cell. Diffusion coefficient distribution histogram for all targets. Black line indicates population distribution before antibody treatment and red line indicates the population distribution after the antibody was treated. The shift in diffusion coefficient is as follows. IgG: 1.95%, CD86: 2.05%, ErbB2: 10.81%, ErbB3: 8.17%, MET: 19.92%, CD44: 8.25%, EphA2: 16.27%, PDGFRb: 11.16%, Integrin5a: 3.53%.


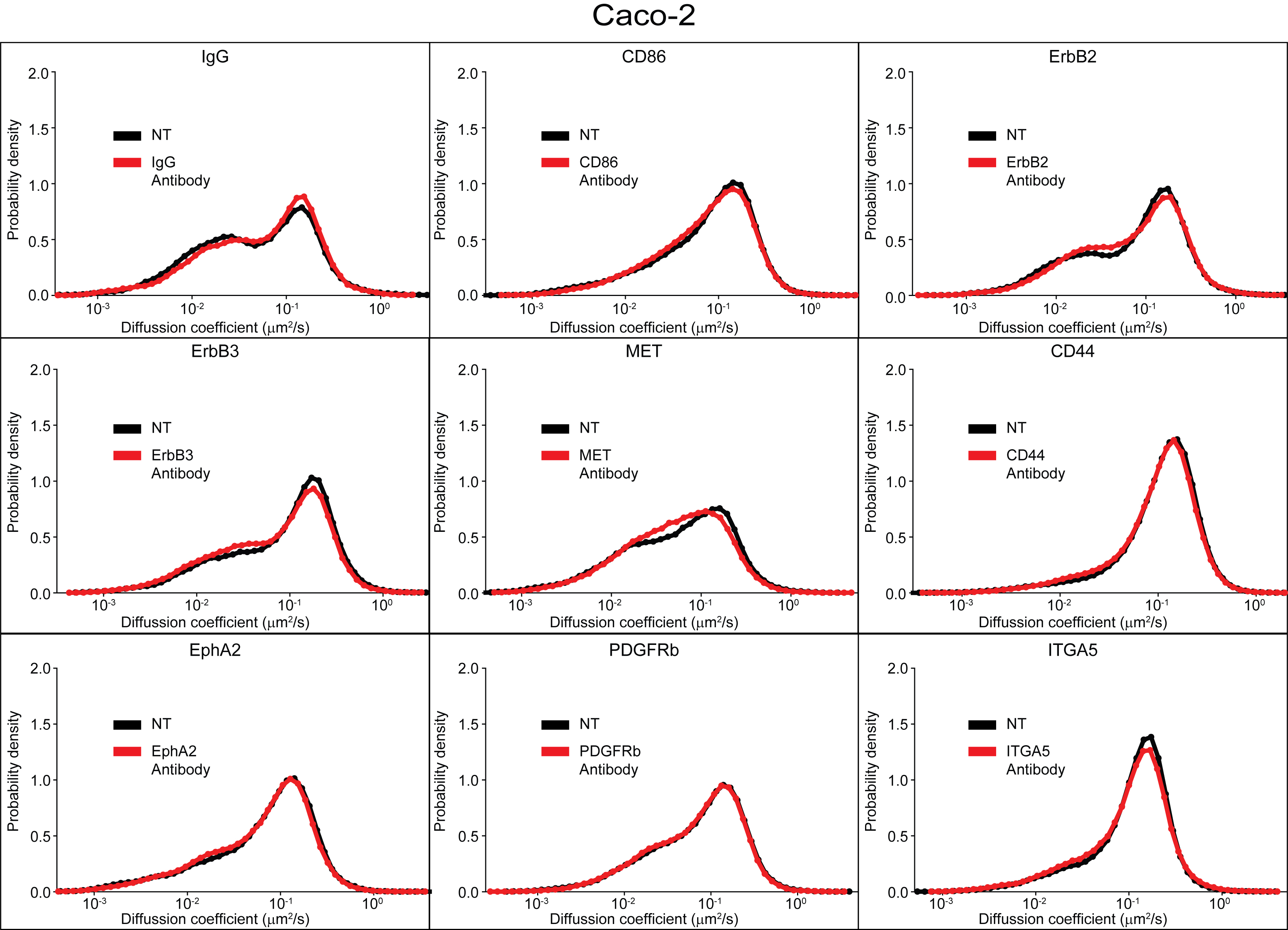


Supplementary Figure 7. Diffusion coefficient distribution for Caco-2 cell. Diffusion coefficient distribution histogram for all targets. Black line indicates population distribution before antibody treatment and red line indicates the population distribution after the antibody was treated. The shift in diffusion coefficient is as follows. IgG: 1.46%, CD86: 3.84%, ErbB2: -1.70%, ErbB3: 3.48%, MET: 11.49%, CD44: 0%, EphA2: 3.17%, PDGFRb: 2.16%, Integrin5a: 3.94%.


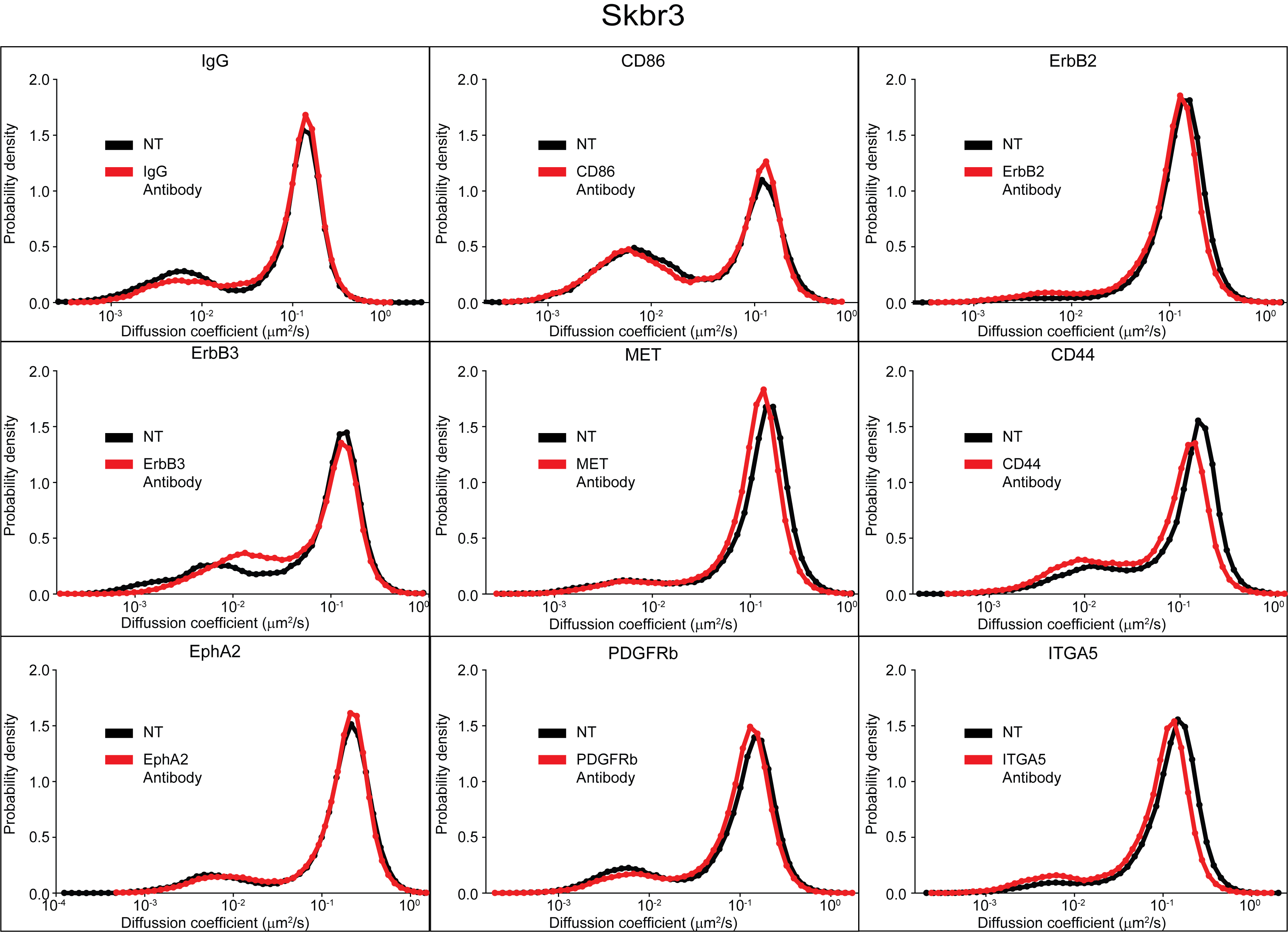


Supplementary Figure 8. Diffusion coefficient distribution for Skbr3 cell. Diffusion coefficient distribution histogram for all targets. Black line indicates population distribution before antibody treatment and red line indicates the population distribution after the antibody was treated. The shift in diffusion coefficient is as follows. IgG: 1.21%, CD86: 2.88%, ErbB2: 10.13%, ErbB3: 5.49%, MET: 13.74%, CD44: 18.27%, EphA2: 2.66%, PDGFRb: 9.30%, Integrin5a: 17.11%.


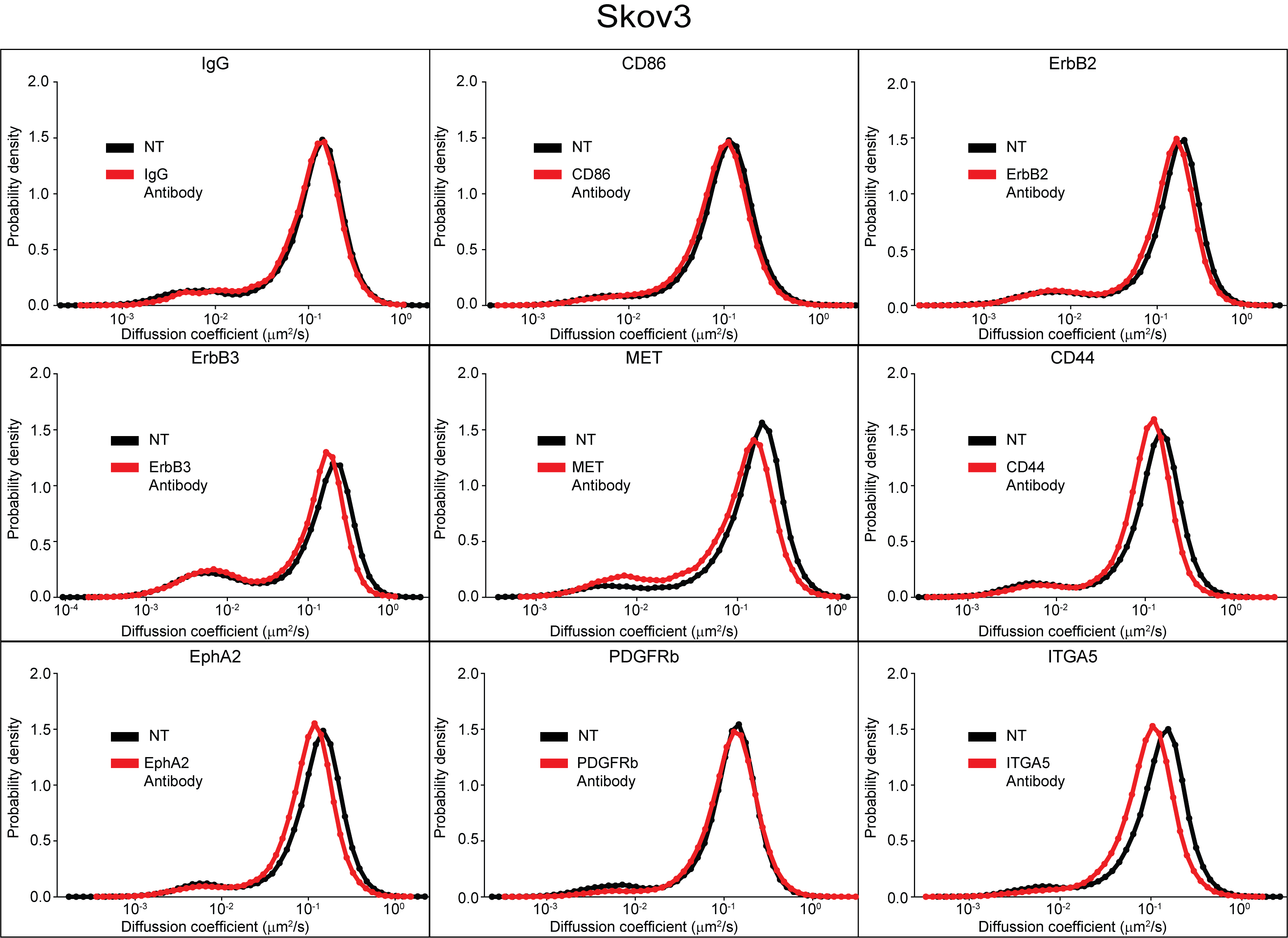


Supplementary Figure 9. Diffusion coefficient distribution for Skov3 cell. Diffusion coefficient distribution histogram for all targets. Black line indicates population distribution before antibody treatment and red line indicates the population distribution after the antibody was treated. The shift in diffusion coefficient is as follows. IgG: 2.31%, CD86: 4.10%, ErbB2: 12.58%, ErbB3: 17.57%, MET: 17.40%, CD44: 18.38%, EphA2: 17.70%, PDGFRb: 2.14%, Integrin5a: 23.37%.

**Supplementary Table 1**. Receptor expression information in each cell lines.

|  | A431 | A549 | Caco-2 | Skov3 | Skbr3 |
| --- | --- | --- | --- | --- | --- |
| ErbB2 | ^1^ | ^2^ | ^3^ | ^4^ | ^5^ |
| ErbB3 | ^6^ | ^7^ | ^8^ | ^9^ | ^10^ |
| Met | ^11^ | ^12^ | ^13^ | ^14^ | ^13^ |
| CD44 | ^15^ | ^16^ | ^17^ | ^18^ | ^19^ |
| EPHA2 | ^20^ | ^21^ | ^22^ | ^23^ | ^22^ |
| PDGFRb | ^24^ | ^25^ | ^26^ | ^27^ | ^28^ |
| Integrin5a | ^29^ | ^30^ | ^31^ | ^32^ | ^33^ |

**References**

1. Kong, A., et al. HER2 Oncogenic Function Escapes EGFR Tyrosine Kinase Inhibitors via Activation of Alternative HER Receptors in Breast Cancer Cells. *PLoS One* 3(2008)

2. Castorina, A., et al. Expression profile of ErbB receptor's family in human alveolar type 2-like cell line A549 exposed to hexavalent chromium. *Toxicol. In Vitro* 22:541-7 (2008)

3. Li, N., Lu, H., Chen, C., Bu, X. and Huang, P. Loss of fatty acid synthase inhibits the "HER2-PI3K/Akt axis" activity and malignant phenotype of Caco-2 cells. *Lipids Health Dis.* 12:83 (2013)

4. Magnifico, A., et al. Tumor-initiating cells of HER2-positive carcinoma cell lines express the highest oncoprotein levels and are sensitive to trastuzumab. *Clin. Cancer Res.* 15:2010-21 (2009)

5. Fehling-Kaschek, M., Peckys, D. B., Kaschek, D., Timmer, J. and Jonge, N. Mathematical modeling of drug-induced receptor internalization in the HER2-positive SKBR3 breast cancer cell-line. *Sci. Rep.* 9:12709 (2019)

6. Khan, I. H., et al. Microbead arrays for the analysis of ErbB receptor tyrosine kinase activation and dimerization in breast cancer cells. *Assay Drug Dev. Technol.* 8:27-36 (2010)

7. Yu, Z., et al. Downregulation of both EGFR and ErbB3 improves the cellular response to pemetrexed in an established pemetrexed-resistant lung adenocarcinoma A549 cell line. *Oncol. Rep.* 31:1818-1824 (2014)

8. Ramsauer, V. P., et al. Muc4-ErbB2 complex formation and signaling in polarized CACO-2 epithelial cells indicate that Muc4 acts as an unorthodox ligand for ErbB2. *Mol. Biol. Cell* 17:2931-41 (2006)

9. Xu, F., et al. The outcome of heregulin-induced activation of ovarian cancer cells depends on the relative levels of HER-2 and HER-3 expression. *Clin. Cancer Res.* 5:3653-60 (1999)

10. Davies, S., et al. High incidence of ErbB3, ErbB4, and MET expression in ovarian cancer. *Int. J. Gynecol. Pathol.* 33:402-10 (2014)

11. Pozner-Moulis, S., Pappas, D. J. and Rimm, D. L. Met, the hepatocyte growth factor receptor, localizes to the nucleus in cells at low density. *Cancer Res.* 66:7976-82 (2006)

12. Bray, S. M., et al. Genomic characterization of intrinsic and acquired resistance to cetuximab in colorectal cancer patients. *Sci. Rep.* 9:15365 (2019)

13. Song, N., et al. Cetuximab-Induced MET Activation Acts as a Novel Resistance Mechanism in Colon Cancer Cells. *Int. J. Mol. Sci.* 15:5838-5851 (2014)

14. Wu, C. C., Weng, C. S., Hsu, Y. T. and Chang, C. L. Antitumor effects of BMS-777607 on ovarian cancer cells with constitutively activated c-MET. *Taiwan. J. Obstet. Gynecol.* 58:145-152 (2019)

15. Geng, S., Guo, Y., Wang, Q., Li, L. and Wang, J. Cancer stem-like cells enriched with CD29 and CD44 markers exhibit molecular characteristics with epithelial-mesenchymal transition in squamous cell carcinoma. *Arch. Dermatol. Res.* 305:35-47 (2013)

16. Penno, M. B., et al. Expression of CD44 in human lung tumors. *Cancer Res.* 54:1381-7 (1994)

17. Vazquez-Iglesias, L., et al. Surface expression marker profile in colon cancer cell lines and sphere-derived cells suggests complexity in CD26(+) cancer stem cells subsets. *Biol Open* 8(2019)

18. Zhou, J., et al. CD44 Expression Predicts Prognosis of Ovarian Cancer Patients Through Promoting Epithelial-Mesenchymal Transition (EMT) by Regulating Snail, ZEB1, and Caveolin-1. *Front. Oncol.* 9:802 (2019)

19. O'Brien, S. K., et al. Breast cancer cells respond differentially to modulation of TGFbeta2 signaling after exposure to chemotherapy or hypoxia. *Cancer Res.* 75:4605-16 (2015)

20. Larsen, A. B., et al. Activation of the EGFR gene target EphA2 inhibits epidermal growth factor-induced cancer cell motility. *Mol. Cancer Res.* 5:283-93 (2007)

21. Li, N., et al. Chimeric Antigen Receptor-Modified T Cells Redirected to EphA2 for the Immunotherapy of Non-Small Cell Lung Cancer. *Transl. Oncol.* 11:11-17 (2018)

22. Liu, Z., et al. YSA-conjugated mesoporous silica nanoparticles effectively target EphA2-overexpressing breast cancer cells. *Cancer Chemother. Pharmacol.* 81:687-695 (2018)

23. Thaker, P. H., et al. EphA2 expression is associated with aggressive features in ovarian carcinoma. *Clin. Cancer Res.* 10:5145-5150 (2004)

24. Perrone, F., et al. PDGFRA, PDGFRB, EGFR, and downstream signaling activation in malignant peripheral nerve sheath tumor. *Neuro Oncol.* 11:725-36 (2009)

25. Reinmuth, N., et al. Combined anti-PDGFRalpha and PDGFRbeta targeting in non-small cell lung cancer. *Int. J. Cancer* 124:1535-44 (2009)

26. Wehler, T. C., et al. PDGFRalpha/beta expression correlates with the metastatic behavior of human colorectal cancer: a possible rationale for a molecular targeting strategy. *Oncol. Rep.* 19:697-704 (2008)

27. Mundhenke, C., et al. Imatinib mesylate inhibits C-kit and PDGF mediated cell growth in vitro in ovarian cancer. *J. Clin. Oncol.* 22:227s-227s (2004)

28. Alexander, P. B., et al. Distinct Receptor Tyrosine Kinase Subsets Mediate Anti-HER2 Drug Resistance in Breast Cancer. *J. Biol. Chem.* 292:748-759 (2017)

29. Nakaoka, H. J., et al. Mint3-mediated L1CAM expression in fibroblasts promotes cancer cell proliferation via integrin alpha5beta1 and tumour growth. *Oncogenesis* 6:e334 (2017)

30. Zhang, J., et al. Cytological effects of honokiol treatment and its potential mechanism of action in non-small cell lung cancer. *Biomed. Pharmacother.* 117:109058 (2019)

31. Sakharov, D., et al. Towards embedding Caco-2 model of gut interface in a microfluidic device to enable multi-organ models for systems biology. *BMC Syst. Biol.* 13:19 (2019)

32. Gao, Q., et al. Heterotypic CAF-tumor spheroids promote early peritoneal metastatis of ovarian cancer. *J. Exp. Med.* 216:688-703 (2019)

33. Haenssen, K. K., et al. ErbB2 requires integrin alpha5 for anoikis resistance via Src regulation of receptor activity in human mammary epithelial cells. *J. Cell Sci.* 123:1373-82 (2010)
